# Soundwatch: Eighteen years of monitoring whale watch vessel activities in the Salish Sea

**DOI:** 10.1371/journal.pone.0189764

**Published:** 2017-12-22

**Authors:** Elizabeth Seely, Richard W. Osborne, Kari Koski, Shawn Larson

**Affiliations:** 1 The Whale Museum, Friday Harbor, Washington, United States of America; 2 University of Washington, Olympic Natural Resources Center, Forks, Washington, United States of America; Institute of Deep-sea Science and Engineering, Chinese Academy of Sciences, CHINA

## Abstract

The Soundwatch Boater Education Program is a vessel monitoring and public education outreach program. Soundwatch has been run by The Whale Museum (TWM) during the whale watch season (May through September) in the Haro Strait Region of the Central Salish Sea since 1993. Data collection has been in a consistent manner since 1998 and is presented here. The program compiles data on vessel types and vessel interactions with marine mammals with a focus on the Southern Resident killer whale (SRKW), *Orcinas orca*, which was listed as endangered under the U.S. Endangered Species Act (ESA) in 2005. The primary goal of the Soundwatch program is to reduce vessel disturbance to SRKWs and other marine wildlife through the education of boaters on regional, local and federal guidelines and regulations and the systematic monitoring of vessel activities around cetaceans. Since 1998, the number of active commercial whale watching vessels has increased over time; ranging from a low of 63 in 1999, to a high of 96 in 2015. In addition, the number of vessel incidents or violation of regulations and guidelines has also increased; ranging from a low of 398 in 1998 to a high of 2621 in 2012. Soundwatch collected data on 23 incident types, some remaining the same over the 18-year data set and some changing over time. The most common incidents over the 18 years were “Within 880 m of Lime Kiln” and “Crossing the path of whales”. The numbers of people kayaking near whales also significantly increased since 2004 with the incident “kayaks spread out” with a significantly increasing trend making it difficult for whales to avoid vessels. These results suggest a need for further outreach for effective education and enforcement of whale watching guidelines and regulations in the Central Salish Sea.

## Introduction

Killer whales or orcas, *Orcinas orca*, are one of the most widely distributed marine mammals, found worldwide in every ocean [1, 2]. They are most abundant in nearshore colder waters but also occur, at lower densities, in tropical, subtropical, and offshore waters [3, 4]. Killer whales are highly social animals that occur primarily in stable matriarchal social groups (pods) that range in size from two to dozens of animals [5, 6]. Larger groups, containing several hundred individuals occasionally form, are called super pods, and are considered temporary groupings [5, 6]. Three distinct types in the Northeast Pacific are recognized and named for their movement patterns: resident, transient, and offshore [7, 8]. The three types also differ in morphology, ecology, behavior, and genetics [8–13]. All three types are sympatric, being found in the same area and with partially overlapping home ranges but they are not known to breed, socially interact or socially communicate with one another [8, 12, 14]. The resident and transient types have multiple populations or family groups within their range. All three have different diet preferences with offshore orcas feeding on fish and sharks, transient orcas feeding on marine mammals and resident orcas feeding primarily on fish, particularly king or chinook salmon, *Oncorhynchus tshawytscha* [15–18]. The name resident killer whales suggest they stay within local home ranges year- round, yet they are known to travel seasonally far from home [19, 20].

The most studied resident killer whale population in the world is the Southern Resident killer whales (SRKW) in the Salish Sea region of Washington State, U.S.A. and British Columbia, Canada [21]. The Southern Resident killer whales are comprised of three pods: J, K, and L pods [5, 19]. They are considered one "stock" under the U.S. Marine Mammal Protection Act (MMPA) and one "distinct population segment" under the U.S Endangered Species Act (ESA) [20, 21]. The SRKW population size has hovered around 80 whales since 2005, a decline from its estimated historical size of approximately 200 during the late 1800s [21]. The lowest recorded population level was 67 in 1971 due to the live-capture of SRKWs for oceanarium display beginning in the late 1960s causing an estimated 30% decrease in the population [22]. Due to several factors including their sustained small population size, qualification as a distinct population segment, prey scarcity, exposure to organic pollutants, vulnerability to oil spills, and increased exposure to vessel traffic and noise, the SRKW population was listed as endangered under the ESA in 2005 [20, 21].

The Whale Museum (TWM) was founded in 1976 with the focus to study, conserve and promote the conservation of the SRKW population in the Salish Sea. The museum, which opened in 1979, is in Friday Harbor on San Juan Island and is a 501(c)(3) nonprofit organization. The museum’s ongoing mission is to promote stewardship of whales and the Salish Sea ecosystem through education and research. The research data collected, compiled and archived by TWM on whale watch and other vessel trends in association with SRKWs was instrumental in helping determine the SRKWs ESA status [20, 23, 24].

The SRKWs are not only an important part of the marine ecology of the Salish Sea but they are also economically important for the region. Whale watching in the Salish Sea has grown to become a $40–50 million industry [25]. The Whale Museum estimates that annually more than 500,000 people go whale watching on commercial and/or private recreational vessels in the trans-boundary waters of Washington and British Columbia [26]. Whale watching provides people with an opportunity to learn about and appreciate marine wildlife. The conservation rationale for whale watching is the theory that as more people become aware of the importance of marine ecosystems and the animals that inhabit them, increasing numbers of them will work to help preserve them [27, 28]. However, it is important that the large numbers of people who watch and enjoy whales and other marine animals in the wild don't disrupt the animals' behavior, environment and life history activities [29–31].

The Soundwatch Boater Education Program was started in 1993 for data collection by TWM as a stewardship activity to provide an educational presence on the water near whale watching activities. Annual data collection by the Soundwatch team began in 1993 but was not systematically collected across years until 1998. In 2004, the staff created data collection protocols with the National Oceanic and Atmospheric Association (NOAA), the Department of Fisheries and Oceans (DFO) from Canada, and the Canadian Straitwatch Program [32]. The goal of the program was to reduce vessel disturbance to SRKW and other marine wildlife. This goal is met by providing systematic monitoring of vessel activities around cetaceans and through boater education on regional guidelines and state/federal regulations from San Juan County, Washington Department of Fish and Wildlife (WDFW), NOAA, and DFO. The data focuses on whale watching vessel trends, vessel incident trends, and private recreational boater education in the Haro Strait region from 1998–2015. The main question we address here is) Have whale watching vessel numbers and vessel incidents from all types of vessels increased over time?

## Materials and methods

### Boats

**Fig 1:**
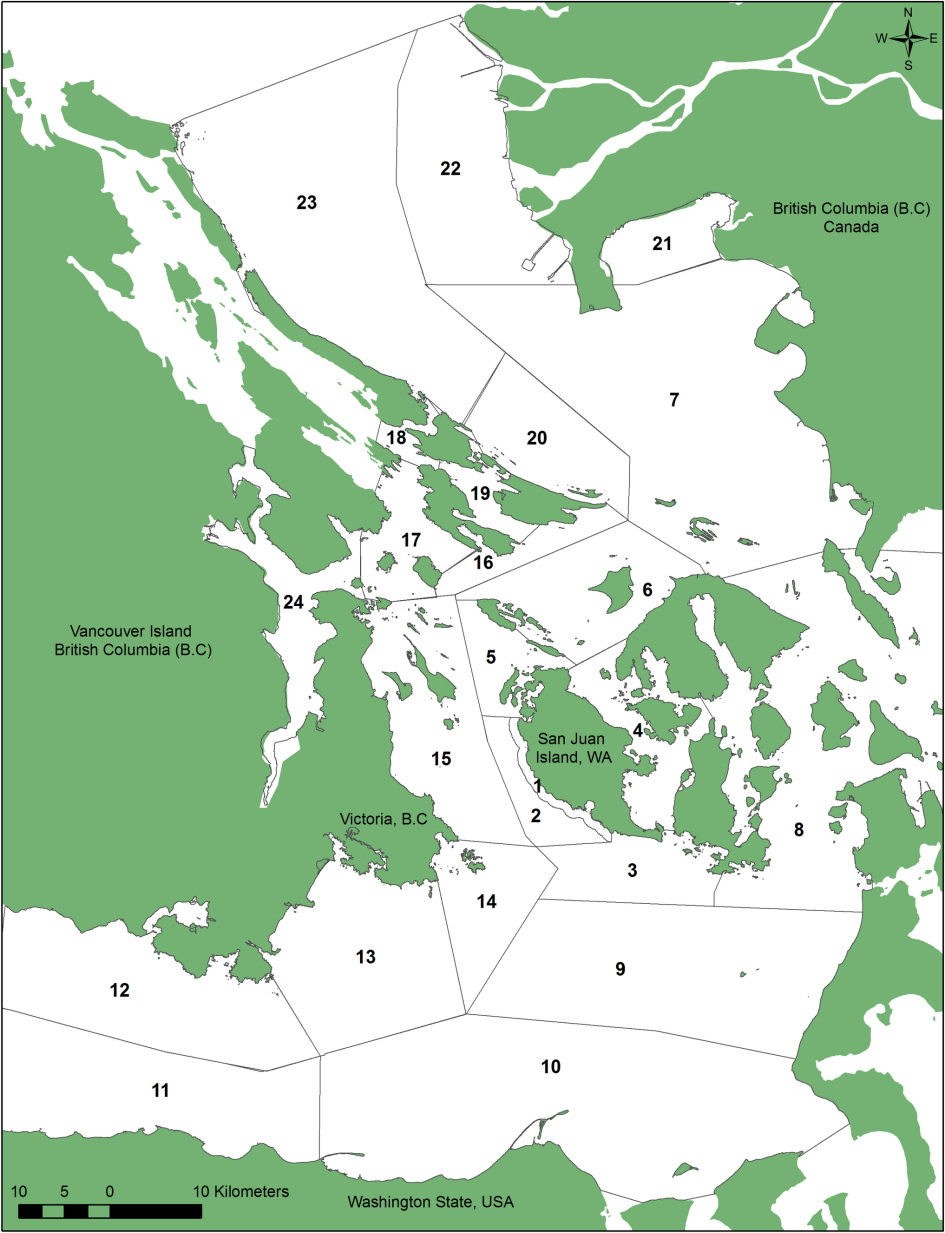
Map of region where Soundwatch collected data.

Soundwatch monitoring vessels were on the water gathering data on vessel numbers, vessel interactions with marine mammals and educating boaters between 1998 and 2015 from May-September, 10am-5pm, during the whale watch season in the Haro Strait region of the Salish Sea (Fig 1). Soundwatch’s effort on the water has been consistent over the years however day to day time on the water may vary slightly due to weather conditions and other variables outside of Soundwatch’s control such as vessel breakdowns. Total hours on the water by Soundwatch each year is reported in Table 1. Soundwatch monitoring vessels have varied through the years and in 2015 was two vessels, a 1993, 5.2 m (17 foot) American Eagle vessel with an outboard four stroke motor with a capacity to hold four people and a 2002, 5.79m (19 foot) Safe Boat with an inboard diesel engine with a capacity for five people. Each vessel was manned by a minimum of an educator/driver and an intern to gather data and may include 2 additional staff, interns or volunteers. All data was collected under the direction of the staff educator/driver. Interns and volunteers help with data entry, photography, etc.

**Table 1:** Soundwatch 1998–2015 all incident types over time.

Incident type	1998	1999	2000	2001	2002	2003	2004	2005	2006	2007	2008	2009	2010	2011	2012	2013	2014	2015
**•Leapfrogging**	143	245	150	5	0	0	0	0	0	0	0	0	0	0	0	0	0	0
**Under power within 0–100 m of whales**	24	31	32	21	13	45	66	95	154	163	170	334	128	194	105	207	219	115
**•Stopped within 0–100 m of whales**														410	210	150	326	180
**•Under power within 100–200 m of whales**														274	262	328	273	131
**•Stopped within 100–200 m of whales**														450	393	127	377	213
**Within 440 m of SJI No-Boat Zone**	155	205	111	90	18	48	31	76	51	54	84	221	106	163	157	53	2	33
**Within 880 m of Lime Kiln**	14	16	13	6	5	18	7	19	12	31	14	77	42	31	52	31	25	33
**Crossing path of whales**	7	25	33	10	10	26	43	38	64	86	56	118	53	61	183	226	208	49
**Chasing/pursuing whales**	10	8	20	10	2	15	20	9	25	31	43	77	32	25	23	22	0	0
**Inshore of whales**	12	229	157	132	48	61	149	172	218	173	297	610	181	325	262	230	228	147
**Airplane within 305 m**	18	16	26	36	37	22	45	38	77	86	113	154	42	94	12	189	56	33
**Within 200 m of National Wildlife Refuge**	15	8	20	3	6	7	8	0	12	3	14	12	8	2	26	22	0	0
**•Other**		8	20	53	36	18	109	105	127	31	28	25	16	0	26	33	10	0
**•Within 220 m of shore; whales present**			26	21	1	3	30	9	26	21	5	20	10	25	52	22	0	0
**•Repositioning within 100 m**			45	36				2										
**•In the Path (formerly Parked in the path of whales)**				129	63	63	137	258	333	184	355	488	245	202	419	396	434	425
**•Fast within 400 m**					6	15	65	95	141	173	156	334	138	157	210	100	199	180
**•1st Approach head on, behind, or on shore**					10	7	9	14	13	21	42	51	42	46	105	16	86	32
**•Kayaks spread out**					2	11	0	9	10	13	14	13	10	11	52	20	26	32
**•Kayaks with whales outside 1/4 SJI Zone (440 m)**					2	3	2	9	8	5	14	23	9	5	26	11	9	0
**•Kayaks paddling w/in 0–100 m**						11	3	9	10	10	14	15	5	10	15	8	10	16
**•Kayaks paddling w/in 100–200 m**														15	26	12	21	16
**•Kayaks parked on headland**															5	1	0	0
**Total Observed Incidents**	**398**	**791**	**653**	**533**	**259**	**373**	**761**	**957**	**1,281**	**1,085**	**1,419**	**2,572**	**1,067**	**2,500**	**2,621**	**2,234**	**2,509**	**1,635**
**Total SW Monitoring Hours with whales present**	**426**	**510**	**426**	**486**	**378**	**312**	**486**	**564**	**516**	**420**	**540**	**420**	**442**	**573**	**306**	**331**	**425**	**393**

### Protocol

Soundwatch documented all vessels in the central Salish Sea near whales and categorizes the commercial vessels as active, occasional, rare or inactive Active vessels were defined as those observed operating more than one day per week during the whale watch and Soundwatch season: May-September. The other categories are one day a week or less, one or two times a month, or once every few months, respectively [24, 25, 33–35]. Soundwatch’s protocol when approaching recreational vessels traveling in known whale or other wildlife areas was as follows: Politely initiate communication with the boater to provide marine wildlife viewing guidelines and regulations as well as to collect information on the vessel and the number of passengers [36]. Throughout the years, Soundwatch has distributed education material that summarized the guidelines and regulations, marine mammal protection areas and whale identification guides. Currently, the primary educational material in distribution was developed by TWM in partnership with local non-government organizations (NGOs) (The Whale Trail, OrcaNetwork, Killer Whale Tales, Whale Scout, etc), international NGOs (BC Sightings Network), federal governments (DFO and NOAA), state government (WDFW), and the Pacific Whale Watching Association (PWWA) resulting in what is now the current “Be Whale Wise Marine Wildlife Guidelines for Boaters, Paddlers and Viewers” that includes the Washington State and U.S. Federal Laws and Regulations for boating around killer whales (S1 Fig and S2 Fig) [36]. Soundwatch also encountered recreational kayakers in a similar manner to communicate the special concerns for kayakers paddling around marine wildlife, distributing the material above as well as another educational guide developed by TWM specifically for kayakers, the “Be Whale Wise Soundwatch Kayak Education and Leadership Program” card (S3 Fig and S4 Fig).

Every time a recreational vessel was contacted, specific contact information was recorded on a Soundwatch Vessel Contact data sheet (S5 Fig). Soundwatch crews recorded the date, time, location, type of vessel contacted, the vessel activity, vessel registration, name, description, port of origin, and number of passengers on board. Soundwatch observers collect a series of vessel operator attributes such as: Why the vessel was contacted; whether they took the information and, if not, why; whether they were aware of the information; their best fit reaction to Soundwatch; whether this vessel has been contacted by Soundwatch before; if there was a Soundwatch observed vessel incident recorded with this vessel before or after contact, if so the time of the incident is recorded; if there were photos of this vessel, planned length of visit to the islands and any other relevant comments (S5 Fig). Soundwatch also collects data on non-motorized recreational vessels such as kayaks and paddleboards within 0.8km of cetaceans and uses the same contact form described above. Data collection on these types of recreational vessels launching specifically from the San Juan County Park was done through a boater self-reporting system required by San Juan County Parks and was incorporated into the Soundwatch database at the end of each season.

Surveys or counts of whales and a count of vessels within one km of whales were collected every 30 minutes using the Soundwatch Vessel Count/Whale Survey data sheet (S6 Fig). Soundwatch recorded whale and all vessel data using a set of standardized vessel activity definitions as well as whale attributes agreed upon by U.S. and Canadian cetacean researchers [5, 11, 37]. Vessels within 0.8 km of whale activity and the cetacean(s) of interest, were counted according to type and vessel activity. Range finding tools such as laser range finders, electronic radar and chart plotters as well as high-power binoculars were used to gauge distances. In all cases, Soundwatch staff were instructed to use the best available data collection techniques and equipment to accurately determine distance to not bias results towards a vessel incident or to include more vessels in a count within 0.8 km of a whale when determining distances. The area of whale presence and boat presence near whales was variable and not limited to 0.8 km, but rather represented the core of individual whales or groups of whales in the immediate area and could range upwards of two kilometers.

Each observed vessel within the count range was categorized according to a vessel type and a specific best-fit vessel activity to describe what the vessel was engaged in. Vessel activity categories included transiting (moving through the area within 0.8 km); whale oriented (moving or stationary whale watching); fishing (moving or stationary with poles or nets in the water); research (engaged in any type of research); enforcement (enforcement vessel in pursuit or engaged with a vessel at the time of the count); acoustic (outside of the count range one km, but in acoustic/visual range); or other (which must be described, such as a rescued vessel in tow, etc.) (S6 Fig).

### Vessel incident observations

Soundwatch recorded vessel incidents on a separate sheet (S7 Fig). Soundwatch used an adaptive management approach (i.e., changing guidelines annually to meet changing vessel/whale conditions). All incidents occurred when any vessel was out of compliance with local/state/federal guidelines and U.S. regulations [23, 36]. To complicate total incident numbers over the years there have been shifts in the types and numbers of vessel incident categories over time. In 2011 there was one new incident category added to reflect the new U.S. federal vessel regulations: vessel within 100–200 m of whales (the second part of the new 2011 regulation; stopped 200–400 m in the path was captured in a previous guideline “parked in the path” incident category). With the addition of this one new incident category, it was now possible to record a single vessel as having two simultaneous incidents when the vessel is observed within 100 m of a whale: 1- within 100 m and 2- within 200 m.

The Soundwatch vessel was included in all vessel monitoring data and was accountable any time it was out of compliance with any guideline and regulations including the updated Washington State and U.S Federal laws. Soundwatch staff recorded every time that the Soundwatch vessel could have possibly been within 400 m ahead or within 0–200 m of killer whales. Since the new 2011 U.S. federal vessel regulations, Soundwatch staff made a more targeted effort to reach as many boaters as possible before those boaters found themselves out of compliance with the new vessel regulations of “In the Path 0–400 m.” To further comply with regulations and guidelines, Soundwatch began operating under its own specific NOAA Research permit in 2012, signified by a yellow permit flag (Permit No. 16160). This allowed for close approaches to whales in some unavoidable circumstances and were reported via permit conditions and annual reporting requirements. In years prior to 2012, Soundwatch educators/drivers operated under NOAA research permits held by various partners to remain in compliance. The majority of the time, the Soundwatch vessel was well over 200 m to the side or beyond 400 m ahead or behind whales to be in the best position to reach on-coming vessels before they encountered whales.

Regression in the Real Statistics Resource Pack software (Release 5.1, www.real-statistics.com) was used to determine significant trends over time.

## Results and discussion

### Number of vessels

**Fig 2:**
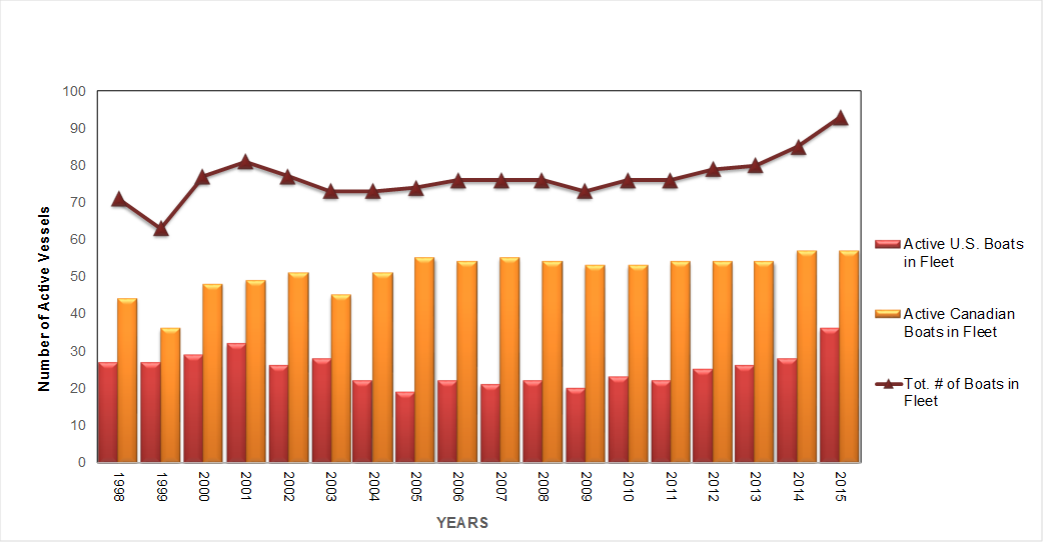
Soundwatch commercial vessel data 1998–2015. Reprinted from [23] under a CC BY license, with permission from [The Whale Museum], original copyright [2015].

Soundwatch's cohesive data collection began in 1998, many years after organized commercial whale watching tours in the Haro Strait region started in the mid 1970's. In 1998, Soundwatch documented 71 active whale watching vessels. Since then, the number of active commercial whale watching vessels dropped to a low of 63 in 1999, and then reached a peak of 93 in 2015 (Fig 2), a significant increase over time (R^2^ = 0.44 P = 0.002).

The active commercial whale watch vessels in the Haro Strait region were composed of approximately equal numbers of U.S and Canadian companies. Over time, the trend has been with more Canadian vessels, totaling 57 active vessels compared to 36 U.S. active vessels. This data includes vessels that are listed as occasional or inactive (93 active and 3 occasional inactive boats). The majority of both U.S. and Canadian commercial companies operating in the trans-boundary waters were members of the PWWA, formerly Whale Watch Operators Association Northwest (WWOAN). Canadian commercial vessels continue to be mostly smaller rigid hull inflatable (RHIB) style vessels, while the U.S. fleet is made up mostly of larger passenger style vessels. However, several Canadian companies have added large passenger style vessels, in addition to existing RHIB vessels, to their company fleets. Additionally, U.S. companies have recently added small cruiser-style vessels to their existing fleet of larger passenger style vessels. The trend recently has been for U.S. companies to operate small, cruiser type vessels, many of them unmarked or minimally identified as commercial whale watching vessels.

**Fig 3:**
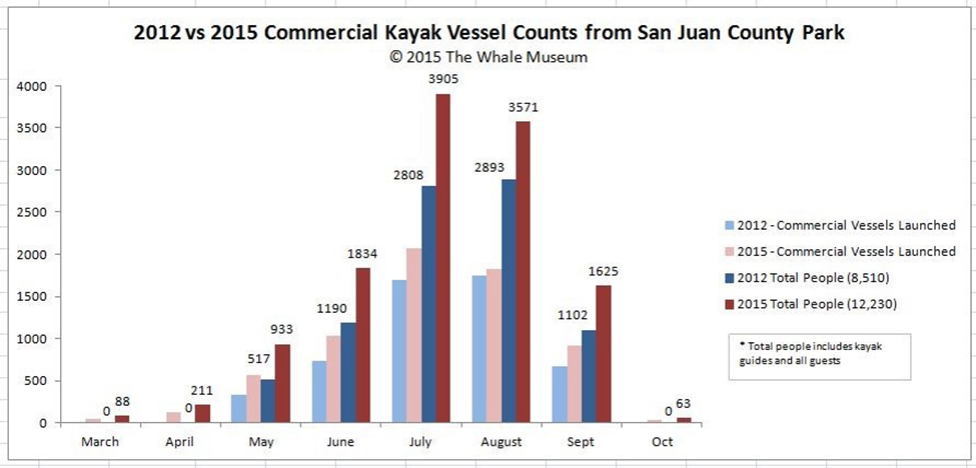
Increase in commercial kayaks participating in whale watching activities. Reprinted from [23] under a CC BY license, with permission from [The Whale Museum], original copyright [2015].

Small commercial vessels such as kayaks, canoes and paddleboards add complexity to the types of vessels observed with whales. In 2015 alone, there were 12,230 reported people kayaking equaling approximately 6,100 vessels with commercial whale watch companies from San Juan County Park. Tracking only kayakers engaged in whale watch activities between 2012 and 2015, there has been a 30% increase in the number of kayakers with commercial whale watching companies (Fig 3) including a corresponding increase in the number of kayak incidents (Table 1). Since 2004, when Soundwatch began tracking kayaks, there has been a significant upward trend in the annual number of commercial kayakers launched from San Juan Island (R^2^ = 0.89 P<0.001).

**Fig 4:**
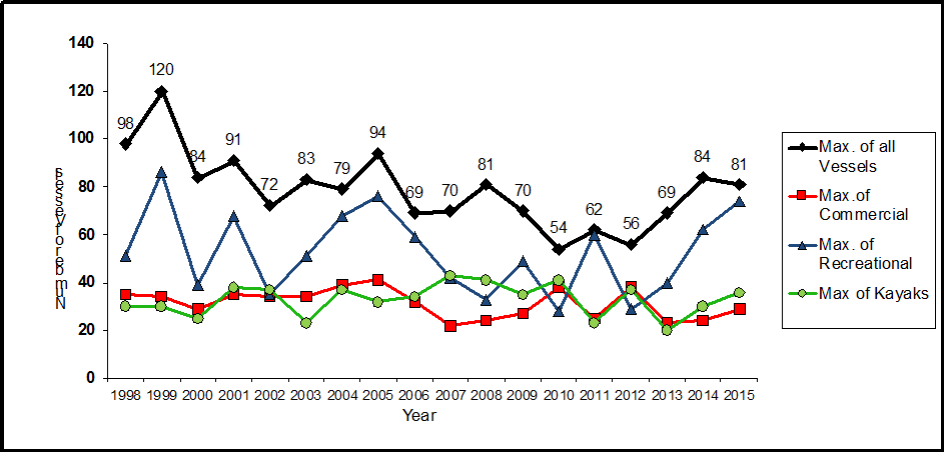
Annual maximums of vessel types accompanying SRKWs May-September 1998–2015. Reprinted from [23] under a CC BY license, with permission from [The Whale Museum], original copyright [2015].

Soundwatch found wide variability in the overall number and types of vessels with whales and their activities on the water. This wide variability is a factor not only of month and time of day, but also due to whale locations overlapping with vessels engaged in a variety of activities such as fishing. For example, from 2011–2015, the first five years after the federal regulations were put into place, the average percentage of vessel activities observed near whales were, 63% whale oriented, 17% transiting, 12% fishing (commercial or recreational), 4% acoustic influence, >2% research, and >1% enforcement or monitoring. Of these activities, the average type of vessels recorded from 2011 to 2015 were; 45% commercial whale watch vessels, 27% private recreational vessels, 8% marine industry (shipping/cargo and commercial fishing), 6% monitoring vessels (Soundwatch), 7% kayaks, 1% government (enforcement and military), and 3% research vessels. The majority (65%) of vessels observed within 0.8 km of whales were engaged in whale watching activities. The highest densities of vessels within 0.8 km of whales occurred in the same areas frequently used by SRKWs, within one kilometer near-shore along the west side of San Juan Island (Fig 1). Over the time-period reported here there has been a significant downward trend of active whale watching vessels within 0.8 km of whales suggesting compliance with whale watching regulations by active commercial companies (R^2^ = -0.37 P = 0.006, Fig 4). The numbers of overall vessels within 0.8 km of SRKWs follows a bi-modal peak at 1100 and 1500 hours reflecting morning and afternoon commercial whale watching trips.

There are several factors that could explain the variability of vessels involved in whale watching activities over the years. First, the vessels are whale oriented and TWM’s SRKW sightings in the Haro Strait vary from year to year [38, 39]. Second, is that SRKWs sometimes spend more time in smaller spread-out groups rather than in tight larger groups thus spreading out the number of whale watching vessels on each groups of whales. This would potentially mean that Soundwatch was with a different group of whales that was out of sight of many of the vessels and therefore would be unable to count all potential vessels whale oriented with SRKWs [24, 36, 40, 41]. Finally, the variability in the number of vessels recorded with whales is also reflected in the variability of other wildlife viewing opportunities. For example, in some years when SRKW sightings have been low, there have been increased numbers of transient killer whales (*Orcinus orca*), minke whales (*Balaenoptera acutorostrata*), humpback whales (*Megaptera novaeangliae*), pacific white-sided dolphins (*Lagenorhynchus obliquidens*), gray whales (*Eschrichtius robustus*), occasional fin whales (Balaenoptera physalus) and steller sea lion (*Eumetopias jubatus*) haul outs, in the region also drawing both commercial and recreational whale watching vessels and kayaks [40–42].

### Vessel incidents

Monitoring hours or effort by Soundwatch on the water varied each year due to a variety of factors such as the number of days SRKWs spend in the Salish Sea, number of days other wildlife is observed in the Salish Sea, vessel maintenance and operability, fuel consumption and weather. The 18-year average numbers of hours on the water was 442 hours, with a low of 306 in 2013 and a high of 573 in 2012, with no notable change over time (R^2^ = 0.05 p = 0.036) (Table 1).

Soundwatch contacted nearly 1,000 recreational vessels each year between 1998–2011 to hand out educational materials. Between 2011–2015 Soundwatch contacted an average of 550 vessels each year. During this time, the average number of people on board each vessel was 3.3, with an average of 1,815 total recreational boaters reached each year for an approximate education of 9,000 boaters over five years. There were a number of reasons for the decrease in vessels contacted during the last five years such as: different priority level for contacting vessels (if they were behaving well Soundwatch didn't contact them right away); Soundwatch didn't try to contact all recreational fishing vessels in high density areas because of the potential for greater disturbance to the whales; less contact for vessels in violation of national wildlife refuge distance; whales more spread out and fewer whale days in the Central Salish Sea; and more enforcement vessel presence around whales. Since 2009, the yearly average of the recreational vessels contacted stated they were unaware of the guidelines and laws for boating around killer whales was 61%.

The top vessel incident types from 1998–2015 were as follows: vessels in the path of whales; vessels motoring inshore of whales; vessels motoring within 100 m of whales; vessels stopped within 100 m of whales; vessels motoring fast within 400 m of whales; and vessels motoring within the 0.5 km voluntary no go zone (Table 1). The incident vessels within 200 m of whales was divided into two categories in 2011: 1- Stopped within 100–200 m of whales and 2- motoring under power within 100–200 m of whales (Table 1). The last two incident types are also among the most common incident types (Table 1). Over the past ten years between 2006–2015, private recreational vessels remain the most likely vessel type to commit all incidents (60%), followed by Canadian commercial vessels (19%), U.S. commercial operators (11%), kayakers (4%), monitoring (including Soundwatch), research vessels (2%), aircraft (2%), and then other (2%).

**Fig 5:**
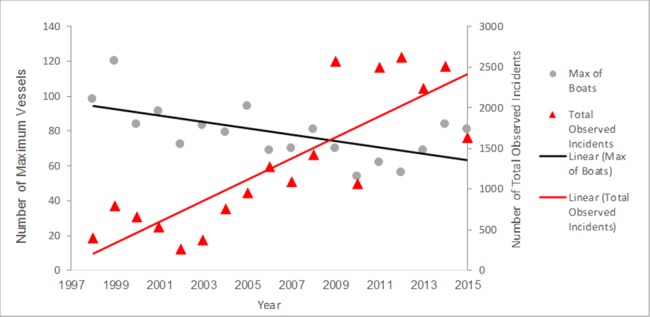
Maximum number of vessels accompanying Orcas and total incidents per year. Reprinted from [23] under a CC BY license, with permission from [The Whale Museum], original copyright [2015].

**Fig 6:**
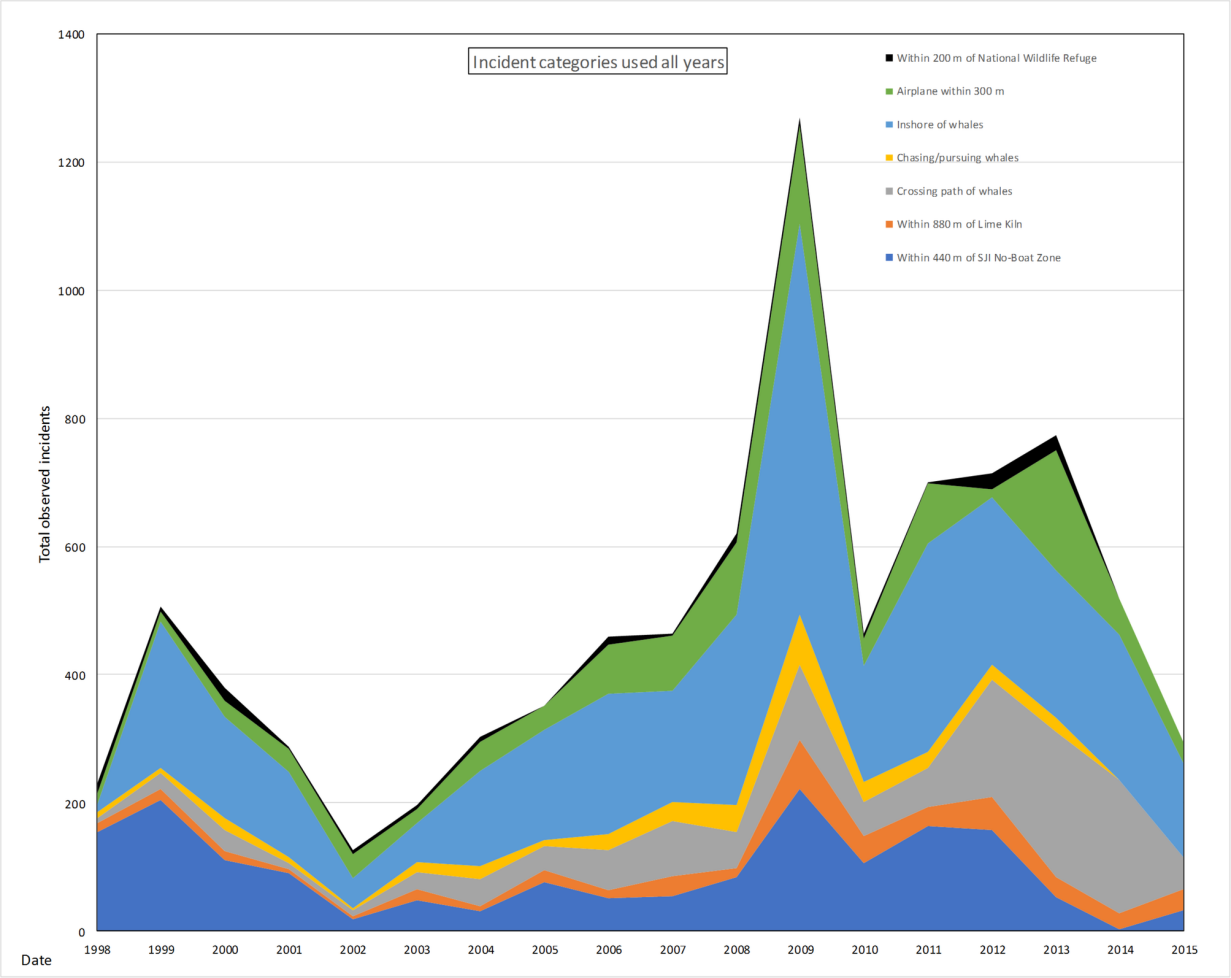
Incident trends from categories used from 1998–2015.

**Fig 7:**
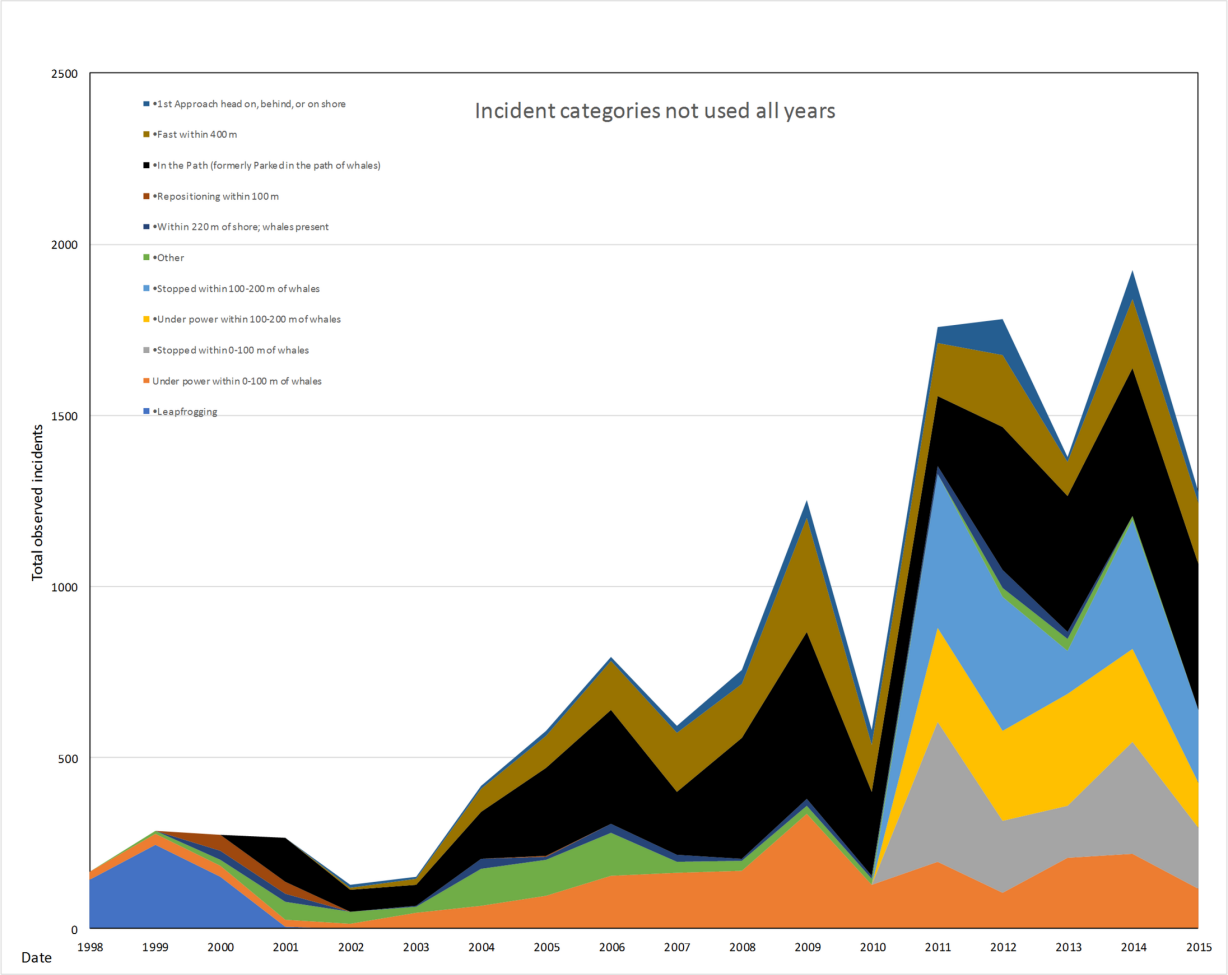
Incident trends from most categories not used all years 1998–2015.

The total numbers of incidents or vessels operating contrary to guidelines and regulations has increased in the past 18 years from 398 in 1998 to 1635 in 2015 and with the highest number of vessel incidents recorded in 2012 with 2621 (Fig 5). The numbers of incidents types used consistently all years increased significantly over time (R^2^ = 0.24 p = 0.04, Fig 6). The numbers of incident types not used all years also increased significantly over time (R^2^ = 0.77 p<0.001) (Fig 7).

Incidents types used consistently over the 18 years peaked in 2009, the year with the largest number of total incidents (1269), followed by smaller peaks for incident categories between 2011–2013 (701, 715, and 773 respectively) with many incident categories decreasing in 2015 (295) (Fig 6). In 2015, there was a noticeable change in the distribution and travel behavior of the whales, possibly a reason for the decreased count of total incidents [43].

Totals for incident categories not used all years started going up significantly in 2009 (1303), decreased in 2010 (603), and then remained high from 2011–2015 (1906, 1431, 1990, and 1340 respectively) (Fig 6).

Not every incident category increased over time. Only two incident categories used all years significantly increased over time: Within 880 m of Lime Kiln (R^2^ = 0.34, p = 0.01) and In the path of whales (R^2^ = 0.53, p<0.001). Incident types used all years that were not significantly increasing were: Within 440 m of SJI no boat zone (R^2^ = 0.04, p = 0.40), Chasing/pursuing whales (R^2^ = 0.03, p = 0.48), Inshore of whales (R^2^ = 0.20, p = 0.06), Airplane within 300 m (R^2^ = 0.21, p = 0.054), and Within 200 m of National Wildlife Refuge (R^2^ = 0.002, p = 0.84). The significantly increasing trends; Within 880m of Lime Kiln, and Within the path of whales were likely due to the fact that this is an area with the highest numbers of vessels engaging in whale watching activities.

There were more incident categories significantly increasing over time in the categories not used all years. Those that increased over time were: Under power within 0–100 m of whales (R^2^ = 0.51, p<0.001); In the Path (formerly Parked in the path of whales) (R^2^ = 0.63, p<0.001); Fast within 400 m (R^2^ = .37, p = 0.02); and 1st Approach head on, behind, or on shore (R^2^ = 0.42, p = 0.01). Categories not used all years that did not increase were: Repositioning within 100 m (R^2^ = 0.21, p = 0.09); Stopped within 0–100 m of whales (R^2^ = 0.32, p = 0.85); Under power within 100–200 m of whales (R^2^ = 0.47, p = 0.30); Stopped within 100–200 m of whales (R^2^ = 0.02, p = 0.31); Other (R^2^ = 0.08, p = 0.25); Within 220 m of shore whales present (R^2^ = 0.00, p = 0.92) (Fig 7). The categories with increasing trends were boaters trying to position themselves to view whales to get closer for those ionic whale shots, or to see the whales swimming underwater, or due to not knowing what the laws and guidelines are, or were recreational fishermen with lines in the water and ultimately failing to give whale’s space and a free path when moving.

**Fig 8:**
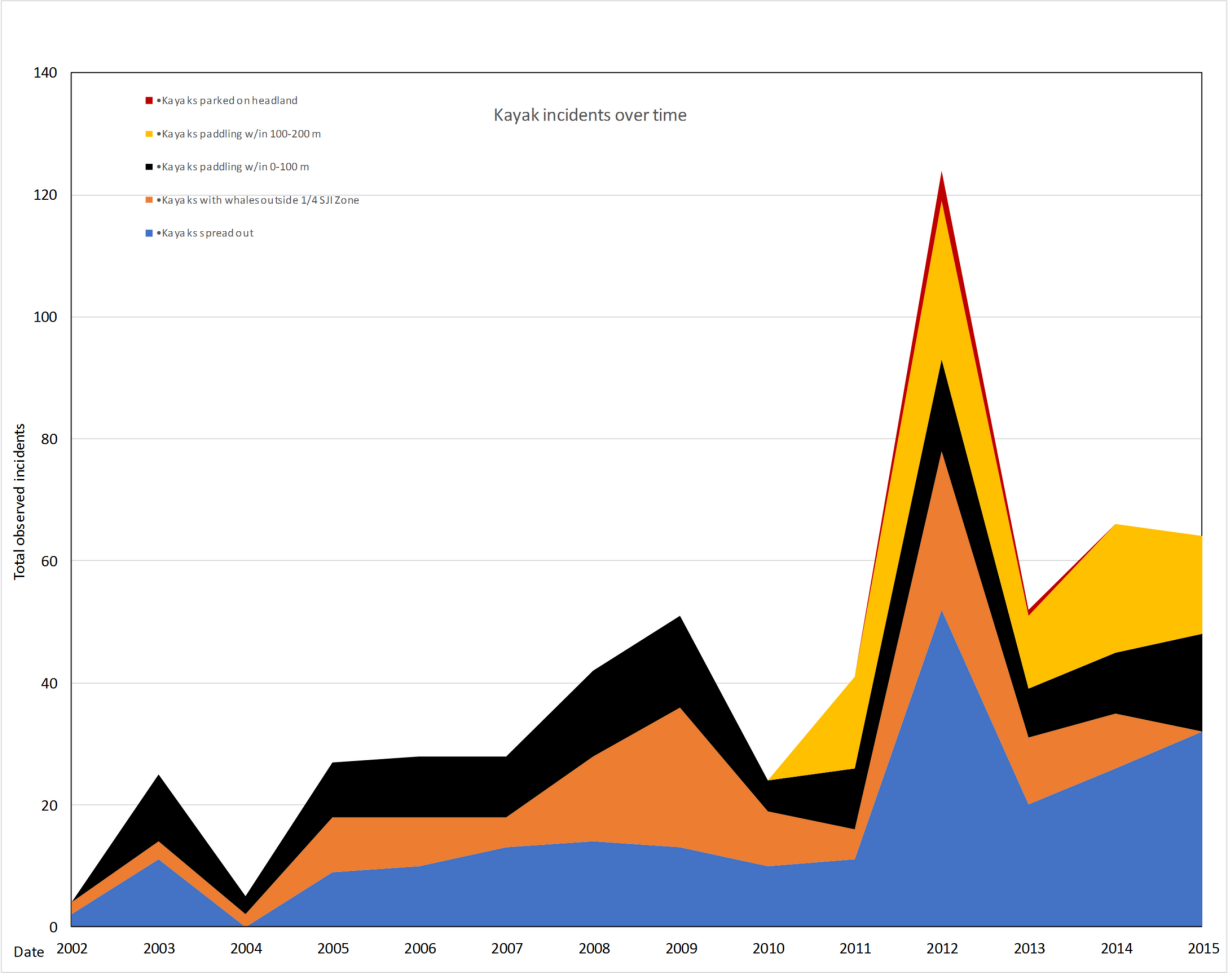
Kayak incident categories from 2002–2015.

Incidents with kayakers with whales have increased since 2002 and they have their own associated incident categories. By far the year with the highest number of kayaking incidents was 2012 (124) followed by relatively high numbers of incidents between 2013–2015 (52, 66 and 64 respectively) (Fig 8). Only one category showed an increasing trend, kayaks spread out with whales (R^2^ = 0.45, p = 0.01). This may be due to the increasing numbers of kayakers spreading out from shore to see whales more closely and then failing to come together or “raft up” when whales are present to decrease potential disturbance.

Between 1998 and 2015 specific incident types involving vessels maneuvering to get the best view of the whales as possible increased over time. Private recreational vessels observed within 0.8 km of whales were more likely to be observed committing vessel incidents than commercial whale watch vessels within the same 0.8 km of the same group of whales. This is thought to be due to a number of reasons including but not limited to: private recreational boaters spending more overall time being ‘whale oriented’ (watching whales) rather than transiting through the area, private recreational boaters being unaware of cetaceans in the area while transiting, and private recreational boaters being unaware of the guidelines and/or regulations. The majority of vessels and vessel incidents were most likely to occur along the west side of San Juan Island (Within 880 m of Lime Kiln) because spatially this is where SRKWs are most often seen. Further education of boaters may be needed whether it’s on the water as buoys or on navigational chartplotters or additional requirements by local ports to increase the shore based education of boaters. Soundwatch is an educational presence on the water, however without stricter enforcement, the education may not be reaching enough boaters that frequent the Central Salish Sea to decrease or stabilize the numbers of incidents over time.

**Fig 9:**
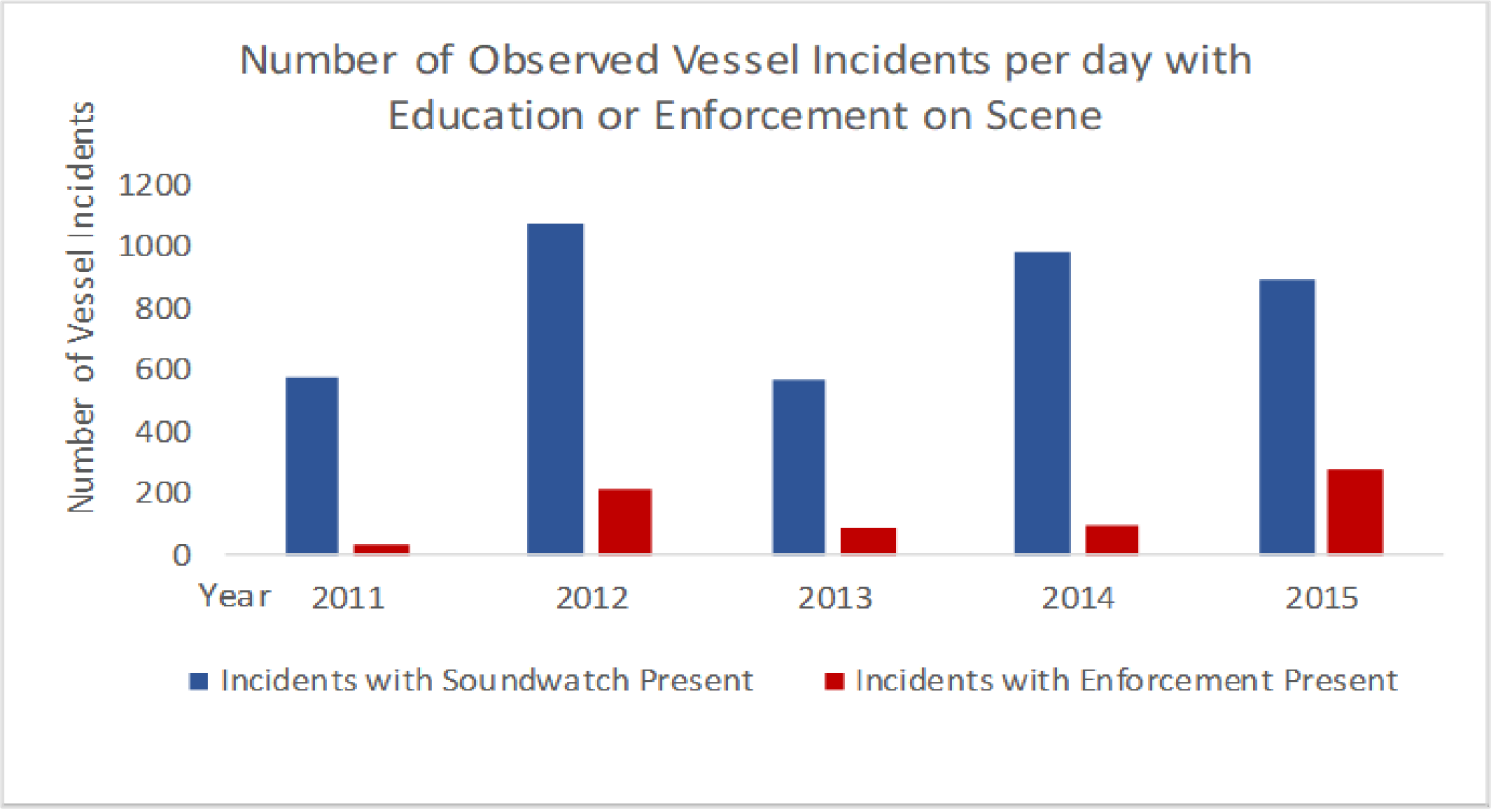
Enforcement presence versus Education presence on scene with whales from 2011–2015.

In 2015, 47% of the vessels contacted by Soundwatch responded that they were unaware of the whale watching guidelines and/or vessel regulations around whales. This percentage is close to previous years suggesting the outreach, education and enforcement efforts may need to be increased. Despite the numbers of overall whale watching vessels has remained consistent (Fig 5). In the past decade, the following regulation incidents have increased: In the path of whales; vessels within 0–100 m and vessels between 100–200 m stopped or under power (new regulations since 2011). While guidelines incidents, such as Fast within 0.4 km, Inshore of whales and Within the 0.4 km Voluntary No Go Zone, are decreasing. Private recreational vessels observed within 0.8 km of whales were more likely to be observed committing vessel incidents than commercial whale watch vessels within the same 0.8 km of the same group of whales. The majority of vessels and vessel incidents are most likely to occur along the west side of San Juan Island (Within 440 m of Lime Kiln) because spatially this is where SRKWs are most often seen. These are the vessels most often contacted by Soundwatch. The effectiveness of the Soundwatch monitoring vessel versus an enforcement vessel on scene with whales can be seen in Fig 9. In the presence of an enforcement vessel, all vessel incidents show a dramatic decrease, indicating that vessels may be more complaint with the full-time presence of an enforcement vessel. Further education of boaters is needed whether it’s on the water as buoys or on navigational chart-plotters or additional requirements by local ports to increase the shore based education of boaters. Soundwatch is able to reach boaters on the water however without stricter enforcement, the education is not reaching many of the boaters that frequent the Central Salish Sea.

Whale watching is a popular activity in the Haro Strait region and the total number of people engaged in whale watching activities, approximately 500,000 people, is likely an underestimate. The commercial whale watch industry (majority PWWA members) do not report individual or total annual numbers of passengers engaged in whale watching in the Haro Strait region [34,37]. To gain a more accurate understanding of the total number of people that have or are currently watching whales from vessels in the region over the last ten years, a targeted trans-boundary whale watching survey would need to be conducted [23] similar to what was undertaken when the industry was in its infancy [27,34,37].

Whale watching has both positive and negative effects. Positive effects of whale watching are the increased empathy people who directly view these charismatic animals feel after their wildlife experience. Negative effects are the vessels disturbing whales through their proximity potentially changing the animals’ basic life history activities such as resting, feeding, social interactions and breeding [29–32, 43–47]. In fact, it is due to the strong scientific evidence of vessels disturbing whales that the regional U.S. and state boating regulations were put into place [19]. Although studies of stress hormone levels from fecal samples collected from SRKWs showed no significant correlation between numbers of vessels and stress levels, it showed the cumulative effect of vessel traffic on the SRKW stress response, particularly during years of relatively low salmon numbers [48]. In addition, data on SRKW activity budgets suggest that SRKWs rest less frequently during daylight hours now than in the 1970s when there were far fewer vessels [14, 47]. The ideal for the health of the whales in the region would be a balanced approach with regulations that limit the time, numbers and types of vessels allowed to engage in whale watching activities and to implement a regulatory safe zone or slow zones and/or "no go" zones. These two activities along with consistent and increased enforcement of federal, state and local vessel boating regulations and guidelines would allow the SRKWs to rest, forage and socialize more often and would allow people the continued opportunity to be inspired by and enjoy the SRKWs.

## Conclusions

Soundwatch's recorded vessel and incident trends from 1998–2015 show increases in both boats and kayaks engaging in whale watching as well as increases in some incident types. Long-term increasing trends in incidents particularly those inshore of whales and in the path of travelling whales placing boats near SRKWs demonstrate the need for the continuation and expansion of shore and water-based boater education as well as increased enforcement to accommodate the still growing popularity of wildlife viewing. In addition, vessel regulations commensurate and consistent with U.S. federal regulations in Canada would ensure the whales’ protection as they cross the border multiple times over the course of a single day, thereby helping eliminate some confusion for recreational and commercial vessels. The continued development and implementation of a collaborative U.S. and Canadian effort to manage both commercial and recreational whale watching as well as other vessel traffic near whales is needed to reduce potential threats to the whales from vessel presence, behavior and underwater noise.

## Supporting information

S1 Fig Be whale wise guide front. (JPG)

S2 Fig Be whale wise guide back. (JPG)

S3 Fig Be whale wise KELP front. (JPG)

S4 Fig Be whale wise KELP back. (JPG)

S5 Fig Soundwatch Vessel Contact data sheet. (JPG)

S6 Fig Soundwatch Vessel Count/Whale Survey data sheet. (JPG)

S7 Fig Vessel incident data sheet. (JPG)

S8 Fig Soundwatch observed incident numbers 1998–2015. (XLSX)

S9 Fig Maximum vessel counts from 1998–2015. (XLSX)
