# Supplementary figures and images for: Soundwatch: Eighteen years of monitoring whale watch vessel activities in the Salish Sea
Source: PLoS One. 2017 Dec 22;12(12):e0189764. doi: 10.1371/journal.pone.0189764 (PMC5741222; doi:10.1371/journal.pone.0189764)

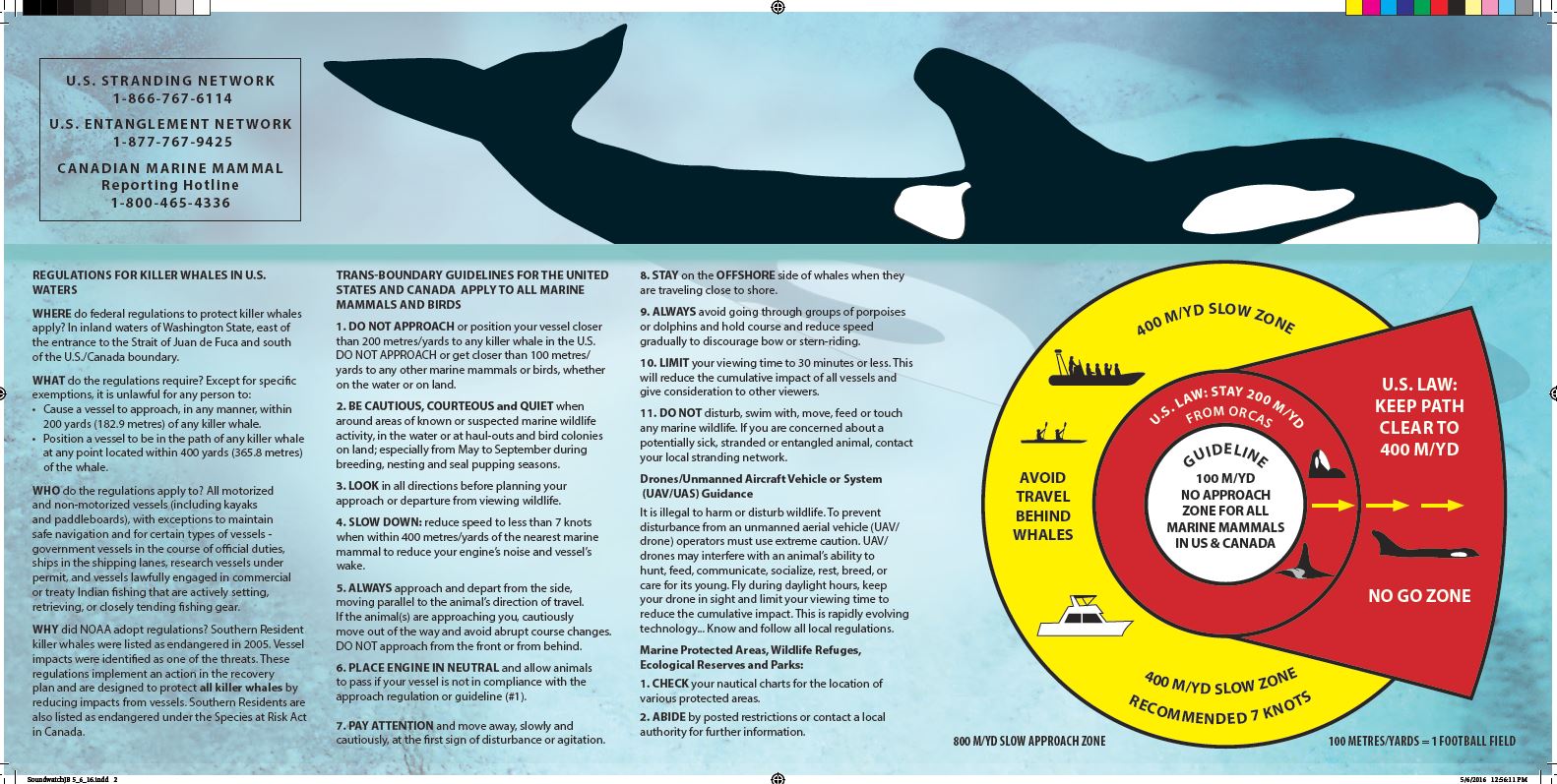

Supplement: S1 Fig — (JPG) [file pone.0189764.s001.JPG]

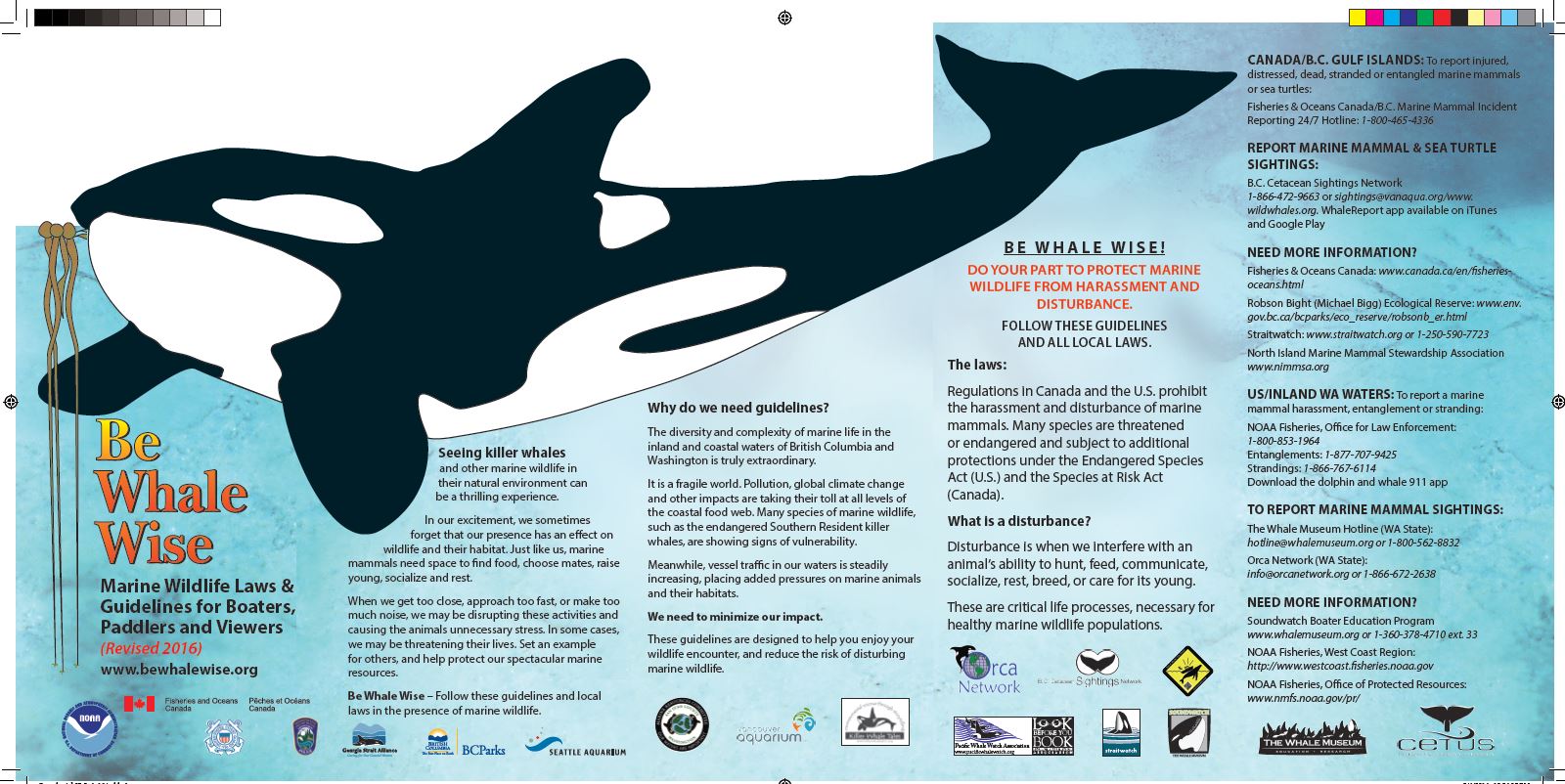

Supplement: S2 Fig — (JPG) [file pone.0189764.s002.JPG]

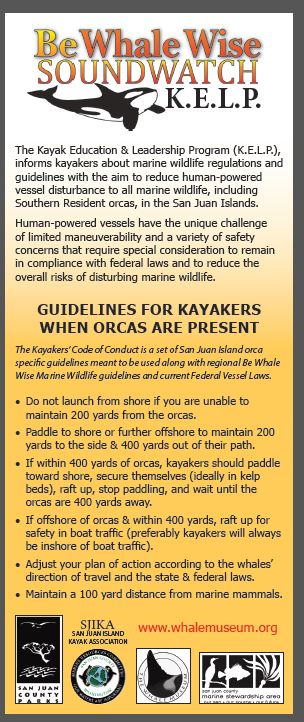

Supplement: S3 Fig — (JPG) [file pone.0189764.s003.JPG]

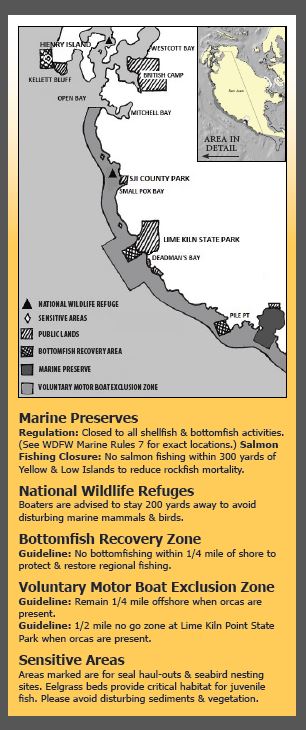

Supplement: S4 Fig — (JPG) [file pone.0189764.s004.JPG]

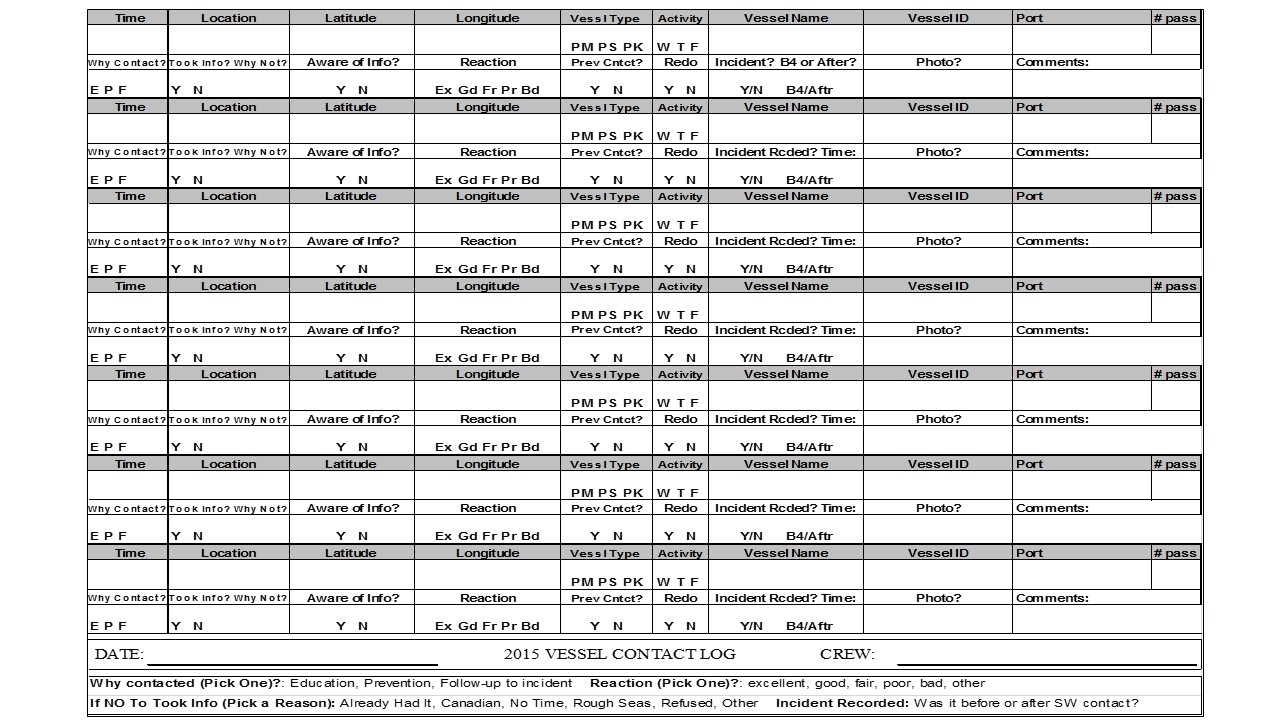

Supplement: S5 Fig — (JPG) [file pone.0189764.s005.jpg]

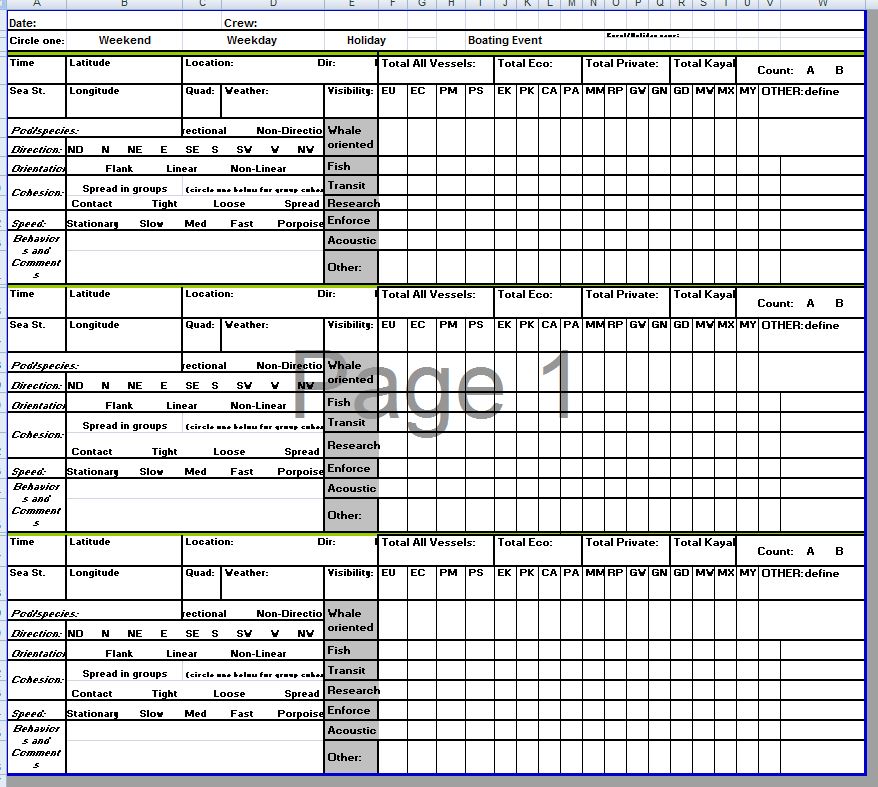

Supplement: S6 Fig — (JPG) [file pone.0189764.s006.JPG]

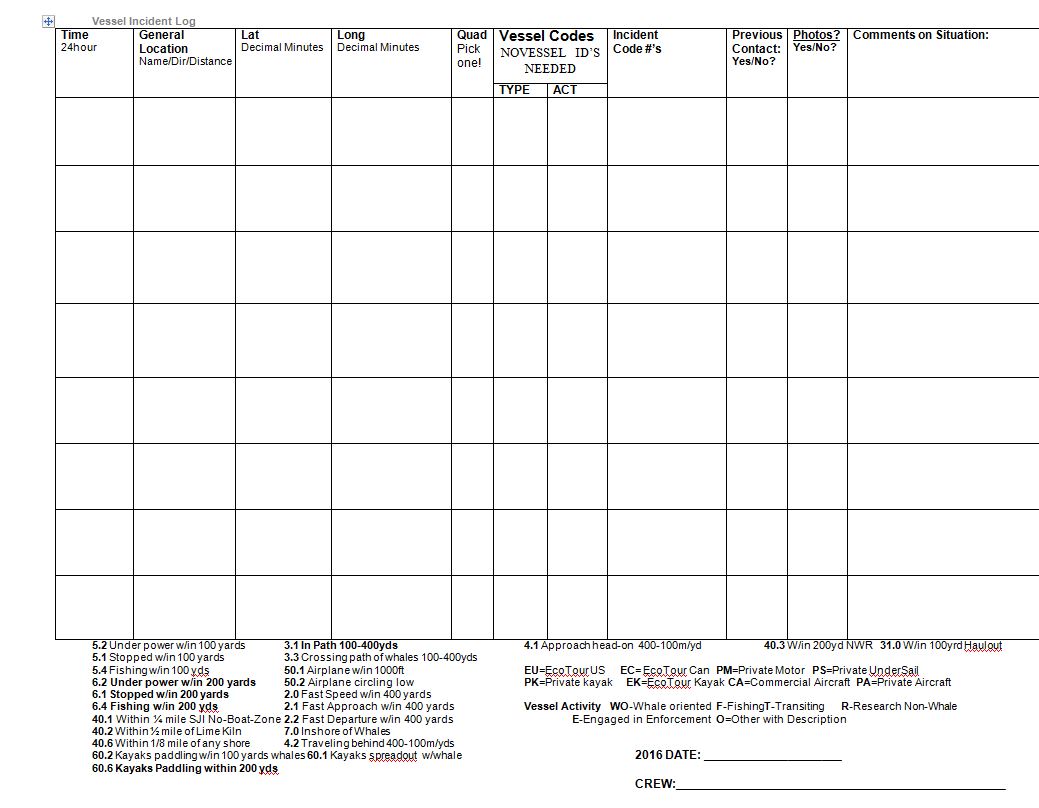

Supplement: S7 Fig — (JPG) [file pone.0189764.s007.JPG]
